# Novel quantitative electroencephalogram feature image adapted for deep learning: Verification through classification of Alzheimer’s disease dementia

**DOI:** 10.3389/fnins.2022.1033379

**Published:** 2022-11-03

**Authors:** Taegyun Jeong, Ukeob Park, Seung Wan Kang

**Affiliations:** ^1^iMediSync, Inc., Seoul, South Korea; ^2^National Standard Reference Data Center for Korean EEG, College of Nursing, Seoul National University, Seoul, South Korea

**Keywords:** electroencephalogram, EEG, QEEG, deep learning, Alzheimer’s disease, XAI

## Abstract

Quantitative electroencephalography (QEEG) analysis is commonly adopted for the investigation of various neurological disorders, revealing electroencephalogram (EEG) features associated with specific dysfunctions. Conventionally, topographies are widely utilized for spatial representation of EEG characteristics at specific frequencies or frequency bands. However, multiple topographies at various frequency bands are required for a complete description of brain activity. In consequence, use of topographies for the training of deep learning algorithms is often challenging. The present study describes the development and application of a novel QEEG feature image that integrates all required spatial and spectral information within a single image, overcoming conventional obstacles. EEG powers recorded at 19 channels defined by the international 10–20 system were pre-processed using the EEG auto-analysis system iSyncBrain^®^, removing the artifact components selected through independent component analysis (ICA) and rejecting bad epochs. Hereafter, spectral powers computed through fast Fourier transform (FFT) were standardized into Z-scores through iMediSync, Inc.’s age- and sex-specific normative database. The standardized spectral powers for each channel were subsequently rearranged and concatenated into a rectangular feature matrix, in accordance with their spatial location on the scalp surface. Application of various feature engineering techniques on the established feature matrix yielded multiple types of feature images. Such feature images were utilized in the deep learning classification of Alzheimer’s disease dementia (ADD) and non-Alzheimer’s disease dementia (NADD) data, in order to validate the use of our novel feature images. The resulting classification accuracy was 97.4%. The Classification criteria were further inferred through an explainable artificial intelligence (XAI) algorithm, which complied with the conventionally known EEG characteristics of AD. Such outstanding classification performance bolsters the potential of our novel QEEG feature images in broadening QEEG utility.

## Introduction

Electroencephalogram (EEG) is an electrical pattern measured at multiple channel locations on the scalp, reflecting cortical activities of the underlying brain regions. Quantitative electroencephalography (QEEG) enables mapping of specific brain functions with the features extracted from digitized EEG through various techniques, such as spectral analysis (Nuwer, 1988).

Raw EEG signals can be decomposed into various waveforms defined by oscillation frequencies through Fourier transform, typically consisting of five frequency bands, designated delta, theta, alpha, beta, and gamma. Delta and theta waves are dominant during the sleeping state, whereas alpha waves (resting state rhythm) are dominant in eyes-closed resting state. Beta waves become dominant when put under stress or during concentration and gamma waves are found when highly alert. However, gamma waves are easily contaminated with a signal artifact that arise from muscle movements (Malik and Amin, 2017; Moini and Piran, 2020).

Quantitative electroencephalography has been employed in the diagnosis of several neurological disorders (Livint Popa et al., 2020), in view of the fact that atypical spectral properties during a certain state correspond to clinically relevant abnormalities. Furthermore, employment of a age- and sex-differentiated normative database can aid in the standardization of the spectral powers, which eliminates variations that arise due to differences in age and sex.

Visualization of EEG spectral powers is crucial for the inspection and diagnosis of abnormalities. Several studies adopt topographic representation, which maps the EEG powers measured at pre-defined channels onto their respective locations over the surface of the scalp. The process can be performed at various frequencies or bands of frequencies, and at different power scales (Yuvaraj et al., 2014).

Although spectral topographies are useful for the visualization of brain activity, it is often difficult to interpret brain functionality as a whole from a single topography which represents spectral power distribution at a specific frequency band. Thus, several topographies at various ranges of frequencies are required to provide a more complete description of brain functionality. Due to this, the use of topographies as feature images for the training of deep learning algorithms becomes problematic seeing as how multiple topographies are required to fully describe a single class label. Therefore, the present study developed a novel QEEG-derived feature image which is capable of overcoming the disadvantages of prior QEEG-based feature sets. The novel feature image sufficiently holds both spatial and temporal information with high resolution and is fully adapted for the training of deep learning algorithms.

Alzheimer’s disease (AD), which the present study focuses on for the verification of the novel feature image, is an irreversible neurodegenerative disorder which is associated with the formation of beta amyloid plaques or neurofibrillary tangles resulting from the dysfunction of microtubule-associated protein tau. The plaques form inside or outside of neurons in the brain, progressively destroying neurons and shrinking the brain, resulting in a gradual decline in cognitive function (Iqbal et al., 2005; Murphy and LeVine, 2010). Patients are usually diagnosed as having AD dementia (ADD) if they are incapable of independent daily living and exhibit imaging β-amyloid plaques and/or tauopathy above a certain threshold, which is most evident in positron emission tomography (PET) scans (Haller et al., 2020). However, PET scans are highly costly, and many nations lack sufficient access to PET scanners. Hence, there have been previous attempts to screen for ADD through QEEG-based features, which are easier to access. For example, Meghdadi et al. (2021) claims that there are resting state EEG biomarkers that can sufficiently indicate characteristics of ADD and mild cognitive impairment (MCI), with strong emphasis on the spectral band theta and alpha. Porcaro et al. (2022) also attempted discrimination of ADD from MCI and an elderly control group using P300 response which is an event-related potential (ERP) component. However, their studies did not employ deep learning algorithms.

Instead, we performed deep learning classification of clinically labeled ADD and non-ADD (NADD) data in the present study, using the novel feature image dataset. Provided that the deep learning model developed in the present study yields a promising classification performance, our novel feature images bear great potential for application in deep learning classification of several other neurological diseases, ultimately resulting in the expansion of QEEG utility.

## Materials and methods

### Electroencephalogram recording and processing

All EEG data employed in the present study were recorded using wet sensor-based Mitsar-EEG systems (Mitsar Co. Ltd., St. Petersburg, Russia) in the eyes-closed resting state, at the 19 channel locations defined by the international 10–20 system (Fp1, Fp2, F7, F8, F3, F4, Fz, T3, T4, C3, C4, Cz, T5, T6, P3, P4, Pz, O1, O2) with linked-ear reference. Electrical impedance was kept at 10 kΩ or below for all channel electrodes. All data were digitized in continuous recording mode for approximately 2–3 min, with sampling rate of 250 Hz which prevents aliasing effects. The ground electrode was located between the AFz and Fz electrodes. All recorded data were re-referenced to a common average reference post-data collection for standardization of the data.

The re-referenced data were further processed using an AI-driven auto-analysis system iSyncBrain^®^ (Ver. 3.0, iMediSync, Inc., Seoul, South Korea) which performs bandpass filtering of signals outside the frequency band of interest (1–45.5 Hz), bad epoch rejection, and independent component analysis (ICA). Electrical patterns detected at multiple channels on the scalp represent a complex weighted sum of several electrical signals, comprised of components originating from electrical sources in the brain, non-stationary noises such as drowsiness or poor contact between electrodes and the scalp, and stationary noises such as eye movement (electrooculography), muscle movement (electromyography), and heartbeats (electrocardiography). Bad epoch rejection aids in elimination of non-stationary noises, while ICA can separate stationary noises as well as help identify their origins (Jutten and Herault, 1991). The ICA components for all data were carefully inspected to assure robust data quality through the removal of artifact components.

### Feature engineering

#### Standardization of the electroencephalogram data

Brain functionalities are known to vary with gender and clearly degenerate with age. To account for this, our study utilized iMediSync, Inc’s normative database ISB-NormDB which holds EEG data of 1,289 healthy control subjects (553 males, 736 females) aged 4.5–81 years old (Ko et al., 2021). The database provides standardized age- and sex-specific features referred to as Z-scores, which are common and statistically robust measures of variation from norms and capture standard deviation. Use of such features can also help reduce a strong dominance of alpha waves, commonly observed in the resting state EEGs of healthy adults measured in the eyes closed condition (Halgren et al., 2019).

The present study utilized sensor level relative power values ranging from 1 to 45 Hz, with a resolution of 0.25 Hz. Z-scores were then calculated *via* reference to ISB-NormDB, which returned 176 Z-score values at 19 electrode locations. We also grouped the spectral powers into eight different frequency bands, namely, delta (1–4 Hz), theta (4–8 Hz), alpha1 (8–10 Hz), alpha2 (10–12 Hz), beta1 (12–15 Hz), beta2 (15–20 Hz), beta3 (20–30 Hz), and gamma (30–45 Hz). Alpha band was subdivided into slower and faster alpha bands (alpha1 and alpha2) since resting state alpha waves are highly related to the cognition status (Ramsay et al., 2021). Beta bands were also subdivided into low, mid, and high beta bands (beta1, beta2, and beta3) in order to dissociate the characteristics of the sensorimotor rhythm (Arroyo et al., 1993).

#### Feature matrix

**FIGURE 1:**
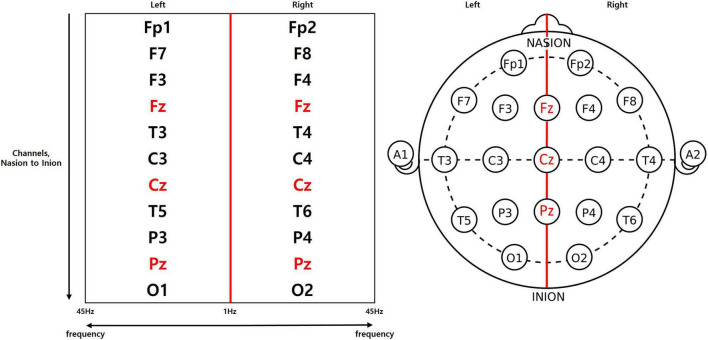
Feature matrix which redistributes the channels into a rectangular arrangement.

Channel locations specified by the international 10–20 system were split into left and right regions, where the central electrodes were taken to belong in both regions. Through rearrangement of the locations into a rectangular format (Figure 1), a single feature matrix was created with the x-axis representing the frequency, and the y-axis representing the channels. Each side of the rearranged matrix consists of 176 frequency bins with a 0.25 Hz resolution, hence the x-axis holds 352 bins in total when both sides are summed. The 19 channels were also rearranged with foremost channels at the top of the matrix and central channels included in both sides. As a result, we acquired a matrix with a shape of 352 by 11.

#### Feature image

**FIGURE 2:**
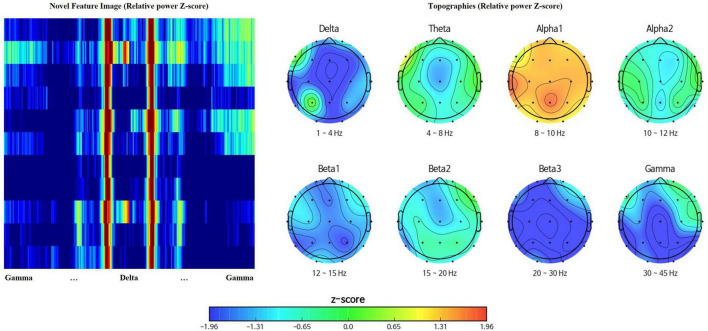
Comparison between the novel feature image and traditional topographies showing clear spatiotemporal resemblance.

Topographies visualize spatial representation of EEG data, at a given frequency band (Arab et al., 2010) while power spectral density presents the amplitude of EEG power per frequency bins describing frequency characteristics (Shim and Shin, 2020). Rearrangement of channels and spectral powers into the above rectangular orientation adopts both the spatial and spectral benefits of topographies and power spectral density. Figure 2 represents the feature matrix visualized as an image with the range of color scale set as -1.96 to 1.96 Z-score, and topographies, yielded from the same EEG data using iSyncBrain^®^.

Multiple feature engineering approaches were applied to the matrix, yielding four types of feature images. The established datasets were used to train and test neural network models. Thereafter, the effects of varying feature images on the classification performance were analyzed.

**FIGURE 3:**
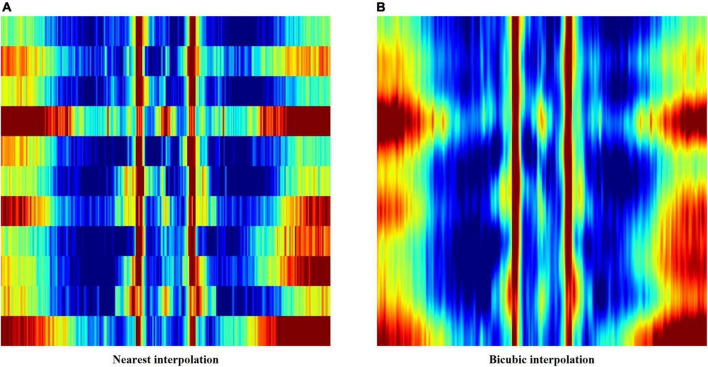
**(A)** Feature image created using nearest interpolation. **(B)** Feature image created using bicubic interpolation from the same electroencephalography (EEG) data.

The first method applied nearest interpolation to the feature matrix, which simply stretches the matrix in the y-direction to match the sizes of the x-axis and y-axis. This yields a square image with a shape of 352 by 352 pixels, with clear edges between consecutive rows and columns (Figures 2, 3A).

In order to smoothen out the edges, bicubic interpolation was applied instead to the feature matrix as the second method. Bicubic interpolation adopts a third degree polynomial to resample data points in both the x- and y- directions, resulting in smoother color transitions (Figure 3B). Equation 1 represents the third degree polynomial used to compute data points through bicubic interpolation. Given that we want to estimate the value of the interpolation surface within the four points (*x*, *x*), (*x*, *y*), (*y*, *x*), and (*y*, *y*), spatial derivatives from the 16 neighboring points to the point (*x*, *x*) are expressed in terms of 16 coefficients *a* (*a*_00_–*a*_33_) using Eq. 1. The interpolation surface *p* (*x*, *y*) can be calculated through the determined values of *a* (Gao and Gruev, 2011).


(1) $$p(x,y)=\sum_{i=0}^{3}\sum_{j=0}^{3}a_{ij}x^{i}y^{j}$$

**FIGURE 4:**
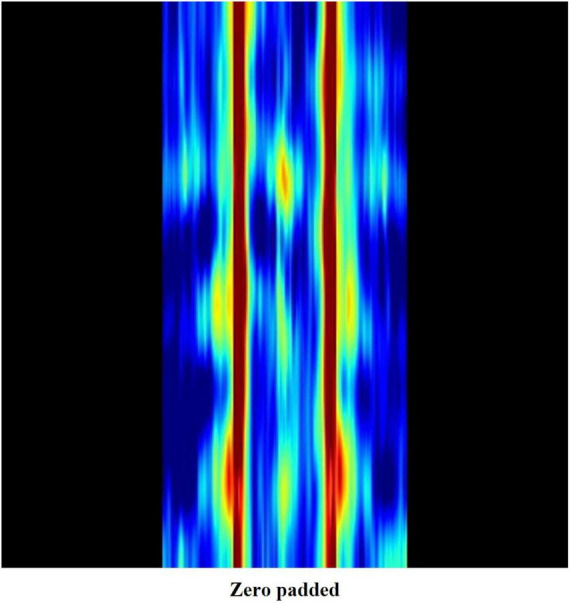
Weight map image where frequencies over 20 Hz are zero padded.

All EEG data were measured in a calm resting state with the subjects’ eyes closed. It is commonly accepted that alpha waves are dominant in such a state, whereas very high frequency waves are easily contaminated by electromyography or external noise. Hence, we created a weight map which zero pads the image regions representing beta3 and gamma frequency bands. In doing so, zero padded pixels appear black when mapped into RGB values (Figure 4), losing their significance as distinguishing features.

**FIGURE 5:**
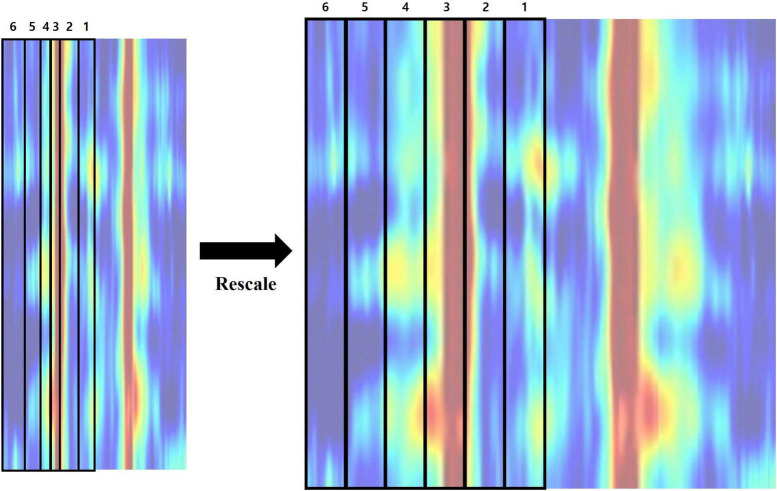
Rescaling the proportions of frequency bands composing the feature image.

Lastly, we rescaled the proportions of frequency bands within the feature image. High frequency regions (beta3–gamma) were first deleted from the feature matrix. A rectangular image with size of 152 by 152 pixels was created by adopting bicubic interpolation, which includes six frequency bands ranging from 1 to 20 Hz (delta–beta2). Each frequency band has a resolution of 0.25 Hz, resulting in a different number of bins. Hence, their proportions are different when visualized as an image, which could influence the effect of a specific frequency band on classification. In order to avoid this, we rescaled each region into matching widths and recreated the feature image (Figure 5 and Table 1).

**TABLE 1:** Size of each frequency band region before and after the rescaling process.

Region	Frequency band	Initial size (x by y)	Rescaled size (x by y)
1	Delta (1–4 Hz)	12 by 152	20 by 240
2	Theta (4–8 Hz)	16 by 152	20 by 240
3	Alpha1 (8–10 Hz)	8 by 152	20 by 240
4	Alpha2 (10–12 Hz)	8 by 152	20 by 240
5	Beta1 (12–15 Hz)	12 by 152	20 by 240
6	Beta2 (15–20 Hz)	20 by 152	20 by 240

Four different image datasets were created using the feature engineering techniques described above and were subsequently used in the deep learning-based classification of ADD and NADD data.

### Classification data

#### Diagnostic criteria

The present study utilized community-based subjective cognitive decline (SCD), MCI, and ADD data received from multiple clinical institutions in South Korea: Chung-Ang University (CAU) hospital; Hanyang university hospital; Inha university hospital; Seoul National University Boramae Medical Center (SNUBMC); Yonsei Severance hospital. Clinical Dementia Rating (CDR) was the main diagnostic criterion along with several other factors, including the clinicians’ independent judgements based on their experiences. CDR is a globally employed method for clinical judgment of the individual’s cognition status through the assessment of six cognitive and behavioral categories (Kim et al., 2017). SCD data is comprised of individuals that met the following criteria commonly used by the Korean AD society (Ho and Yang, 2020): CDR = 0; 60 years old or older; persistent subjective complaints of cognitive decline; completed 6 years of primary school or more; memory test and cognitive test scores below the normative mean within 0–1.5 standard deviations.

Mild cognitive impairment data is comprised of individuals that met the following criteria: CDR = 0 or 0.5; 60 years old or older; persistent subjective complaints of cognitive decline; completed 6 years of primary school or more; memory test and cognitive test scores below the normative mean by more than 1.5 standard deviations; normal performance in activities of daily living (ADL). In addition, clinicians further examined and diagnosed the patients through more comprehensive tests.

Alzheimer’s disease dementia data is comprised of individuals that met the following criteria: CDR ≥ 1; 60 years or older; completed 6 years of primary school or more; memory test and cognitive test scores severely below the normative mean; poor performance in ADL.

Quantitative electroencephalography-based comparisons between the ADD group and NADD group can help identify distinguishing characteristics of ADD and begin to reveal the nature of progression from MCI to ADD. One obvious application for such research includes improving early detection by extending diagnostic signs beyond the current imprecise measure of ADL (Mlinac and Feng, 2016).

#### Dataset establishment

The dataset was segregated into two groups, ADD and NADD. The NADD group consisted of subjective cognitive decline (SCD) and mild cognitive impairment (MCI) data, along with iMediSync, Inc.’s EEG data of healthy individuals from ISB-NormDB (Ko et al., 2021). The inclusion of MCI data in the NADD group was crucial for the identification of significant ADD-specific characteristics that are not observed in the pre-clinical stage of ADD. The final dataset (*N* = 765; 137 ADD, 628 NADD) was established, in which 10% of the data (*N* = 77; 14 ADD, 63 NADD) were randomly selected and excluded as test data for later verification of the developed classification model.

Data imbalance is often inevitable when the classification task involves community-based clinical data collected from clinical institutions. The dataset provided for the present study consisted of significantly less ADD data. Hence, a 9 to 1 train to test ratio was selected, since deep neural networks often require significantly more information for a sufficient training of the network due to its more complex structure in comparison to machine learning algorithms. Although this may raise concerns in chances of overfitting, the significant differences in ADD and NADD QEEG characteristics aid in prevention of overfitting. Thorough verification has also been carried out *via* an explainable artificial intelligence (XAI) algorithm to make certain that the models are not overfitted.

Table 2 describes the established dataset. Age and sex information were not included since age-and sex-standardized Z-scores were employed in the study.

**TABLE 2:** Table describing the dataset used in classification.

		NADD			
		
	ADD	Normal		MCI	Total
		
		NormDB	SCD		
Clinical institutions	137	0	262	142	541
iMediSync, Inc.	0	224	0	0	224
Total	137	224	262	142	765

### Classification model

Transfer learning is a commonly employed method in computer vision research. Building a neural network structure from scratch is time consuming, since various structures must be trained and tested in order to find a structure that outputs a sufficient classification performance. Hence, structures of various pre-established image networks that are known to result in an outstanding image classification performance can be imported and used to train custom datasets (Best et al., 2020). Various image classification tasks, namely, in the detection of objects within images, adopt convolutional neural network (CNN) based image network structures that have already been pre-trained with large-scale data, due to difficulties in the collection of sufficient data needed to train the network from scratch. However, the use of such pre-trained networks is only applicable when classifying similar types of images, in which the network has been pre-trained with. The image datasets established in the present study significantly differ from the dataset that were employed in the pre-training of such image networks. Hence, we adopted only the network structures, training them from scratch.

The objective of the present study is the verification of novel feature images as features for deep learning training, not the development of a state-of-the-art classifier. Hence, complex fine tuning of the image networks had not been performed. Instead, various user-provided sets of hyperparameters were used to train the following pre-established image networks: Alex Network (AlexNet); 16-layer visual geometry group network (VGGNet); 18-layer ResNet. High-performance models that met the set threshold of classification accuracy for each type of image networks were selected and saved. They were then further compared and verified using XAI techniques.

### Image network structures

#### Alex network

AlexNet structure consists of five convolutional layers with selective max pooling layers, and three fully connected layers with 1,000-way softmax at the end. AlexNet employs rectified linear unit (ReLU) activation function instead of the tanh function, which was the standard before the introduction of AlexNet. Dropout regularization method was also applied for the prevention of overfitting (Krizhevsky et al., 2017). Although it showed outstanding classification performance at the time, the network employs large convolution kernels, which results in rapid decline of the feature map size and resolution. Hence, the structure is prone to loss of local features (Xiao et al., 2017; Li et al., 2021).

#### Visual geometry group network

The structure VGGNet was established through the investigation of the effect of network depth on the classification performance. As opposed to large convolutional filters adopted by the AlexNet, VGGNet adopts 3 × 3 convolutional kernels, which enables building of deeper network structure, since there is a slower decline of feature map size and resolution (Simonyan and Zisserman, 2015).

#### Residual network

Residual network (ResNet) structure resolved vanishing gradient problem and overfitting, which are downsides of conventional neural network structures, through the use of residual blocks. A residual block passes the input *x* through the first convolutional layer, followed by ReLU activation and second convolutional layer. The output of the second convolution which is referred to as *f(x)* is added to the original input *x*. The added value *f(x)* + *x* is then passed onto ReLU activation function (He et al., 2016). Although a deeper network with more residual blocks may result in improved classification performance, it is computationally expensive due to the large number of parameters (Okinda et al., 2020).

### Inference of classification criteria: Explainable artificial intelligence

Neural networks are commonly referred to as “black boxes,” since we cannot directly observe or determine the means by which the model classifies unseen data. However, the use of algorithms such as Local Interpretable Model-Agnostic Explanations (LIME) allows us to infer which features the model used for classification. The LIME algorithm creates locally perturbed samples at the borders of a model’s decision function. Each sample carries weights in accordance with the distance between the sample and the initial instance. LIME then learns a local linear predictive model, which is repeated for the multiple borders that exist, but which we cannot discern. Equation 2 represents how the explanations about classification are produced by LIME. ℒ represents local fidelity functions, which is a measure of how unfaithful the prediction model *g* is in approximation of the classification probability *f* in the locally defined space π_*x*_. Ω represents the complexity measures of the prediction model (Ribeiro et al., 2016; Gramegna and Giudici, 2021).


(2) $$\xi (x)=\underset{g\in G}{\text{argmin}}ℒ(f,g,\pi_{x})+\Omega (g)$$

In short, LIME explanations are computed through the minimization of ℒ while keeping Ω low enough for sufficient interpretability of the prediction model. Hereafter, regions that affect the local predictive function are highlighted in order to aid in the understanding of the model’s prediction.

**FIGURE 6:**
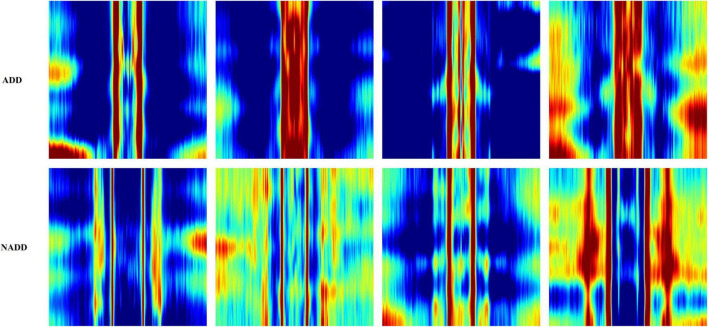
Typical feature images representing the classes Alzheimer’s disease dementia (ADD) and non-Alzheimer’s disease dementia (NADD).

Figure 6 presents typical feature images representing the classes ADD and NADD. The images clearly represent differences in QEEG characteristics between the groups, but we would not be able to identify what exactly the model interprets. In the present study, LIME was employed for clarification of the distinguishable characteristics between groups. The regions which LIME interpreted as top distinguishing features for classification were then used to verify the model’s credibility, in which we take account of clinically known facts in relation to ADD. We further examine the consistency of the selected regions.

### Final model selection

**FIGURE 7:**
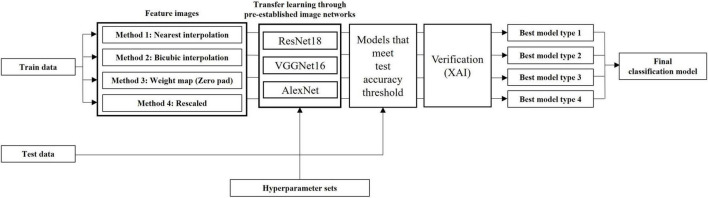
Modeling pipeline which summarizes model yielding and verification processes.

Due to limited computation power, relatively shallower 16-layer VGGNet and 18-layer ResNet were employed in the present study along with the AlexNet. Classification models that showed sufficient accuracy were selected for all types of networks, and images constructed through varying feature engineering methodologies. Each model was inspected through LIME in order to select the best models for each type of image. We then selected the final model which showcased the best classification accuracy. Figure 7 summarizes model yielding and verification processes.

## Results

The same training and test datasets were applied to assess and compare the results of the best classification models, trained using varying types feature images drawn from the aforementioned feature engineering techniques. The 18-layer ResNet image network structure yielded the best classification performance for all four types of images in comparison with other image networks. Table 3 presents the achieved confusion matrices of the best models selected for each network and technique, and Table 4 lists each network’s accuracy, sensitivity, and specificity.

**TABLE 3:** Confusion matrices for selected best models trained with different types of image networks and images constructed through varying feature engineering techniques.

18-layer ResNet					
**1. Nearest**			**2. Bicubic**		
	
	**True ADD**	**True NADD**		**True ADD**	**True NADD**
					
Pred ADD	12	5	Pred ADD	13	4
Pred NADD	2	58	Pred NADD	1	59

**3. Weight map**			**4. Rescaled**		
	
	**True ADD**	**True NADD**		**True ADD**	**True NADD**
					
Pred ADD	14	2	Pred ADD	13	5
Pred NADD	0	61	Pred NADD	1	58

**16-layer VGGNet**					

**1. Nearest**			**2. Bicubic**		
	
	**True ADD**	**True NADD**		**True ADD**	**True NADD**
					
Pred ADD	12	5	Pred ADD	13	5
Pred NADD	2	58	Pred NADD	1	58

**3. Weight map**			**4. Rescaled**		
	
	**True ADD**	**True NADD**		**True ADD**	**True NADD**
					
Pred ADD	13	2	Pred ADD	13	5
Pred NADD	1	61	Pred NADD	1	58

**AlexNet**					

**1. Nearest**			**2. Bicubic**		
	
	**True ADD**	**True NADD**		**True ADD**	**True NADD**
					
Pred ADD	12	5	Pred ADD	13	4
Pred NADD	2	58	Pred NADD	1	59

**3. Weight map**			**4. Rescaled**		
	
	**True ADD**	**True NADD**		**True ADD**	**True NADD**
					
Pred ADD	13	3	Pred ADD	12	5
Pred NADD	1	60	Pred NADD	2	58

**TABLE 4:** Accuracy, sensitivity, and specificity of the established best models trained with different types of image networks and images constructed through varying feature engineering techniques.

18-layer ResNet			

Models	Accuracy	Sensitivity	Specificity
1. Nearest	90.9%	85.7%	92.1%
2. Bicubic	93.5%	92.9%	93.7%
3. Weight map	97.4%	100.0%	96.8%
4. Rescaled	92.2%	92.9%	92.1%

**16-layer VGGNet**			

**Models**	**Accuracy**	**Sensitivity**	**Specificity**

1. Nearest	90.9%	85.7%	92.1%
2. Bicubic	92.2%	92.9%	92.1%
3. Weight map	96.1%	92.9%	96.8%
4. Rescaled	92.2%	92.9%	92.1%

**AlexNet**			

**Models**	**Accuracy**	**Sensitivity**	**Specificity**

1. Nearest	90.9%	85.7%	92.1%
2. Bicubic	93.5%	92.9%	93.7%
3. Weight map	94.8%	92.9%	95.2%
4. Rescaled	90.9%	85.7%	92.1%

**FIGURE 8:**
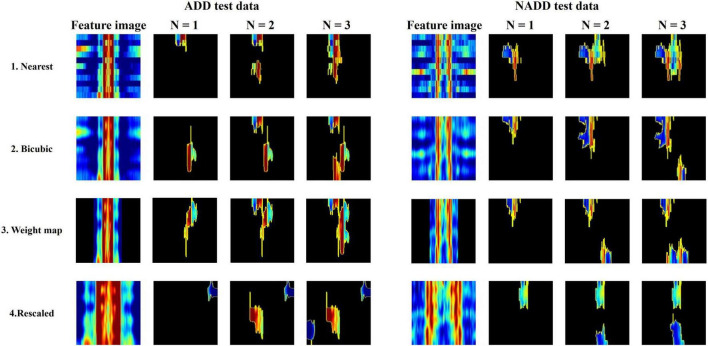
Local Interpretable Model-Agnostic Explanations (LIME) algorithm highlighting summed area of top N regions of importance in classification for a randomly selected Alzheimer’s disease dementia (ADD) and non-Alzheimer’s disease dementia (NADD) test data.

All classification models showed adequate performance, which suggests that the difference in QEEG characteristics between the ADD and NADD groups was significant, and that it was well-represented in our novel feature image. However, the performance of the bicubic model was superior to that of the nearest model, indicating that the block edges among image pixels may negatively affect the classification performance of image networks. The best performance was achieved from the weight map model. This may be because waveforms at faster frequencies are comparatively less important features for classification of ADD. The present assumptions were further verified using LIME, which allowed for comparison among the extracted regions of high importance in classification. A random data index from each group was drawn for comparison among the models (Figure 8). N represents the number of regions that has been added (i.e., *N* = 1 image represents the region of highest importance, and *N* = 3 image represents the sum of the top 3 regions of importance that affected the classification result).

From the regions of importance selected by LIME represented in Figure 8, the classification criteria of the 18-layer ResNet model have been presumed as shown in Table 5.

**TABLE 5:** Final model (18-layer ResNet)’s presumed classification criteria from the regions of importance selected by Local Interpretable Model-Agnostic Explanations (LIME).

Models	ADD classification criteria	Non-ADD classification criteria
1. Nearest	High power of slower waves (delta-theta).	High power of alpha waves and low power of slower waves (delta-theta).
2. Bicubic	High power of slower waves (delta-theta).	High power of alpha waves.
3. Weight map	High power of slower waves (delta-theta).	High power of alpha waves and low power of slower waves (delta-theta).
4. Rescaled	Low power of faster waves (beta 2) and high power of slower waves (delta-theta).	Low power of slower waves (delta-theta).

## Discussion

Through the analysis of 18-layer ResNet classification models *via* LIME, we can infer that the first three models’ classification criteria for ADD involve high power of slower waves, while the rescaled model’s classification criteria involve low power of faster waves. In addition, the rescaled model’s classification criteria for NADD only involved low power of slower waves. Moreover, the bicubic model’s classification criteria for NADD were highly specific to the power of alpha waves. After careful verification with the full test dataset, we deduced that the weight map model yields the best classification performance with foremost robustness.

The objective of the present study was in the establishment and verification of our novel feature image’s capability to contain all useful spatiotemporal information from the EEG data. Since conventional features used for visualization of EEG, namely, topographies are difficult to employ for the training of deep learning models (neural networks), we developed novel feature images from EEG data acquired in a clinical environment and yielded an outstanding deep learning based ADD classification model. The main advantage in use of our feature image is in the visualization of classification criteria through XAI algorithms, which enables more comprehensible analysis on the established classification models.

For further validation, several trials were made to extract regions of importance from test images through LIME. Slight differences were observed for each trial due to the random image sampling algorithm used by LIME. However, each trial showed adequate consistency, hence the classification criteria did not vary. Our findings verified that the power of the slow waves (delta and theta) and that of alpha waves (resting state waves) were crucial factors in differentiating the groups, based on the classification criteria inferred through LIME. The result corresponds to the conventionally known EEG characteristics of ADD, an increment in delta and theta power and a parallel decrement in alpha and beta power in comparison with normal subjects (Jeong, 2004). Moreover, the classification results indicate no signs of overfitting. If the model is overfitted due to an imbalance in the dataset, the model tends to classify data toward the class with larger amount of data. However, the classification model established in the present study resulted in balanced sensitivity and specificity.

In addition, we have made a further comparison between the novel feature image-based classification model established in the present study, with a mini-mental state examination (MMSE) score based method. MMSE is a cognitive mental status examination which are widely in use to aid the diagnosis of several neurological disorders. It consists of short and practical cognitive-specific tests which requires approximately 10 min to complete. The result is scored within the range of 0 which suggests critical deterioration of cognitive functions, to 30 which reflects healthy cognition (Folstein et al., 1975; Perneczky et al., 2006).

**FIGURE 9:**
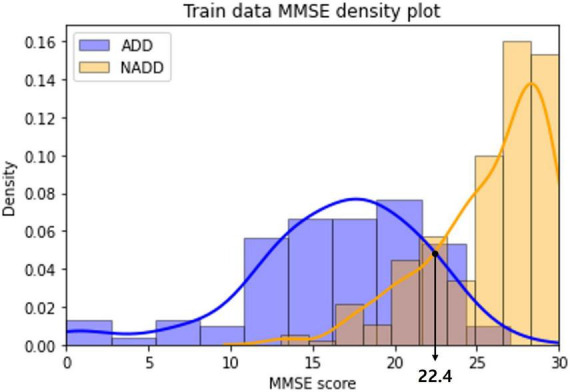
Alzheimer’s disease dementia (ADD) and non-Alzheimer’s disease dementia (NADD) train dataset mini-mental state examination (MMSE) density plot.

The distribution of MMSE scores of the ADD and NADD classes in the train dataset were visualized as a density plot (Figure 9), smoothed off by kernel density estimation.

The classification standard was determined as a cutoff value in accordance with the intersection point of the two density plots: ADD (MMSE ≤ 22); NADD (MMSE > 22). As a result, 1 ADD data and 5 NADD data were misclassified, which is inferior to the classification result of the final model established in the present study.

In addition, we can observe a significant overlap of ADD and NADD MMSE scores, in which signifying that MMSE based classification is less robust. Chapman et al. (2016) also claim that the use of MMSE score cutoffs for the diagnosis of ADD resulted in limited accuracy and were insufficient in the discrimination of MCI from ADD. As opposed to this, the inferred classification criteria for our classification model *via* LIME is based on clear QEEG characteristics that are visually distinguishable.

**FIGURE 10:**
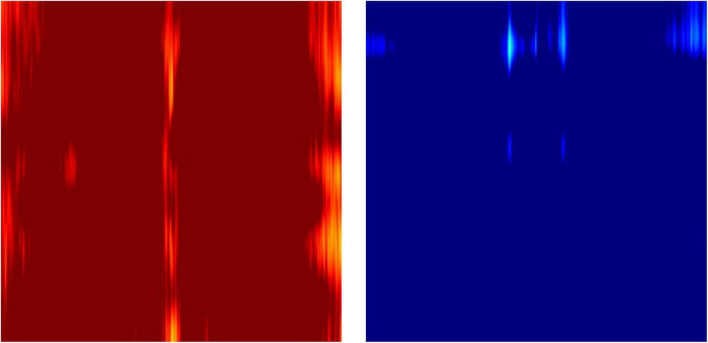
Example outlier images that represent absolute Z-scores of electroencephalography (EEG) power magnitude.

The novel feature images used here only utilized z-scores of relative powers, which conveniently describe the ratio among the powers of waveforms at different frequencies, but not the absolute powers in comparison to the age- and sex- specific normative database. This was due to the fact that individuals have various overall magnitudes of EEG power, which is dependent on thickness of the skull (Hagemann et al., 2008). Therefore, the outliers with excessively strong or weak EEG magnitudes result in almost completely red or blue images when mapped to a -1.96 to 1.96 Z-score color scale (Figure 10).

The downside of the feature matrix extraction method proposed in this study is in the averaging of the time dynamics. In order to account for this, multiple feature matrices can be extracted using a time epoch window that slides over the data and can be concatenated into a 3 dimensional feature matrix. Epoch length used to create each image frame and overlap between consecutive time windows can be altered to yield various resolutions of the time dynamics.

Moreover, the presented feature matrix extraction method is adapted for the spatial representation of 19-channel EEG data since the y-axis of the matrix is essentially a stack of the channels from frontal to rear side regions. Therefore, the presented method might insufficiently represent the spatial information for other commonly used 34- or 64-channel EEG data with several lateral channels. For such cases, the electrodes may be grouped into appropriate regions instead and rearranged accordingly.

The classification model’s outstanding performance and coherence with clinical facts suggest a high potential of the usage of novel feature image as a clinical tool for the diagnosis of several other neurological diseases. Not only limited to this, it can also be utilized for brain-computer interface (BCI) applications, provided that the third of the investigated cases of BCI studies utilized spatiotemporal features (Alzahab et al., 2021). The continual refinement in usage of novel feature images will enhance the ensemble of tools available to more comprehensively leverage the power of QEEG.

## Data availability statement

Inquiries regarding the data used in this present study can be directed to the corresponding author.

## Ethics statement

Ethical review and approval was not required for the study on human participants in accordance with the local legislation and institutional requirements. The patients/participants provided their written informed consent to participate in this study.

## Author contributions

TJ conducted the research and wrote the manuscript. UP supervised the research. SK provided guidance throughout the course of research. All authors approved the submitted version of the manuscript.
